# Exploring and evaluating microbiome resilience in the gut

**DOI:** 10.1093/femsec/fiaf046

**Published:** 2025-04-29

**Authors:** Huimin Zhou, Li Tang, Kristin A Fenton, Xiaobo Song

**Affiliations:** Department of Microbiology, College of Basic Medical Sciences, Dalian Medical University, Dalian, 116044, China; Department of Microecology, College of Basic Medical Sciences, Dalian Medical University, Dalian, 116044, China; Department of Medical Biology, Faculty of Health Sciences, UiT The Arctic University of Norway, Tromsø, 9037, Norway; Department of Medical Biology, Faculty of Health Sciences, UiT The Arctic University of Norway, Tromsø, 9037, Norway

**Keywords:** microbiome resilience, microbiome structure and keystone species, microbe–microbe interplays, host–microbe interactions

## Abstract

The gut ecosystem is closely related to human gastrointestinal health and overall wellness. Microbiome resilience refers to the capability of a microbial community to resist or recover from perturbations to its original state of balance. So far, there is no consensus on the criteria for assessing microbiome resilience. This article provides new insights into the metrics and techniques for resilience assessment. We discussed several potential parameters, such as microbiome structure, keystone species, biomarkers, persistence degree, recovery rate, and various research techniques in microbiology, metagenomics, biochemistry, and dynamic modeling. The article further explores the factors that influence the gut microbiome resilience. The microbiome structure (i.e. abundance and diversity), keystone species, and microbe–microbe interplays determine microbiome resilience. Microorganisms employ a variety of mechanisms to achieve the microbiome resilience, including flexible metabolism, quorum sensing, functional redundancy, microbial cooperation, and competition. Host–microbe interactions play a crucial role in maintaining microbiome stability and functionality. Unlike other articles, we focus on the regulation of host immune system on microbiome resilience. The immune system facilitates bacterial preservation and colonization, community construction, probiotic protection, and pathogen elimination through the mechanisms of immunological tolerance, immune-driven microbial compartmentalization, and immune inclusion and exclusion. Microbial immunomodulation indirectly modulates microbiome resilience.

## Teaser

This review article discusses microbiome resilience from the perspectives of concepts, evaluation parameters and assessment methods, and influential factors of microbiome structure, keystone species, microbial interplays, and host–microbe interactions.

## Introduction

**Figure 1.**
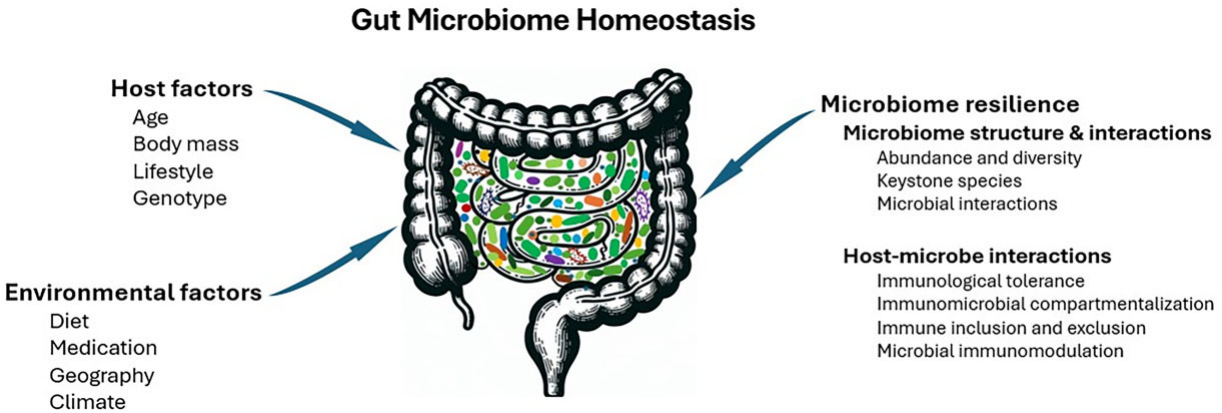
Factors influence gut microbiome homeostasis. Environmental factors and host factors are considered external factors that affect the microbial community balance, while microbiome resilience is considered an internal factor that maintains microbiome stability.

The microbiome is regarded as “the entire habitat, including the microorganisms (bacteria, archaea, lower and higher eukaryotes, and viruses), their genomes (i.e. genes), and the surrounding environmental conditions” (Marchesi and Ravel 2015). There are many habitats in the human body, such as gastrointestinal tract, skin, respiratory tract, genitourinary tract, and each has distinct physio-chemical properties and microbial communities (Berg et al. 2020). Human microbiome is generally present in a state of dynamic equilibrium, with a group of core strains remaining stable over long-time scales and some strains fluctuating over short-time scales in both numbers and proportions (Coyte et al. 2015, Hildebrand et al. 2021). The gut microbiome is remarkably constant in healthy adults. Faith et al. showed more than 70% of the same bacterial strains persisted within one year, and many of them unchanged over the following four years. A number of strains were found dwelling in an individual even for decades (Hildebrand et al. 2021). The gut microbiome exhibited similarities among family members and across geographic regions (Faith et al. 2013). The stable microbiome fits best a power-law function, a mathematical function to determine heterogeneity (spatial aggregation) and stability (temporal aggregation) of the population abundance (Ma 2015). Although the gut microbiome appears to be resilient in healthy humans, it can be influenced by multiple environmental host-microbial factors. Numerous studies have shown environmental factors, such as antibiotics, probiotics, and diets, have strong impacts on the disruption or resurrection of the gut microbiome (Palleja et al. 2018, Ng et al. 2019, Schwartz et al. 2020). Host factors also affect gut microbiome structure, such as host age, body mass, health conditions, exercise frequency, lifestyle, and habits (Rinninella et al. 2019). Lately, the host genotype has become noticeable in constructing and shaping the microbiome (Qin et al. 2022b). Besides the influence from the surrounding conditions, microbiome itself plays a vital role in maintaining gut microbiome homeostasis, which is a stable equilibrium state of microorganisms within the human gastrointestinal tract (Sommer et al. 2017, Fassarella et al. 2021). In this review, we will explore microbiome resilience, with emphasis on the concept, assessment methods, influential factors, and mechanisms in ecology, microbiology, and immunology (Fig. 1).

### Concept of microbiome resilience

There are many ways to define resilience. In ecology, resilience generally refers to the capacity of an ecosystem to withstand (ecological resilience) or recover from disturbances (engineering resilience) (Philippot et al. 2021). Regarding microbiome resilience, Shade et al. defined it as the recovery rate of a microbiome after disturbances. They distinguished between resilience and resistance (insensitivity to disturbance) when evaluating the stability of a microbial community (Shade et al. 2012). On the other hand, Hildebrand et al. defined microbiome resilience as the persistence of a microbial community within a host, family, and geographic region (Hildebrand et al. 2021). Nowadays, microbiome resilience is defined as the overall capability of a microbial community to resist or recover from perturbations to its original state of balance (Sommer et al. 2017, Dogra et al. 2020, Philippot et al. 2021). Resilience is an intrinsic property of a microbial community, which is determined by the constituent microorganisms and the surrounding environmental conditions. Specifically, the gut microbiome resilience is determined by the quantity and quality of constituent microorganisms, microbial activities, microbe–microbe interplays, and host–microbe interactions (Lozupone et al. 2012, Fassarella et al. 2021). Microbiome resilience is critical to maintaining gut microbiome homeostasis, in which microorganisms can function optimally and the hosts are able to exert effective immune protection, thereby benefiting overall human health. Long-term disruptions of gut microbiome homeostasis have been linked to a variety of health disorders and diseases, such as obesity, inflammatory bowel disorders (IBD), diabetes mellitus, hepatic steatosis, metabolic liver disease, and several types of cancers (Lloyd-Price et al. 2019, Frost et al. 2021, de Vos et al. 2022). However, the cause–effect relationship between microbiome imbalance and the disorders or diseases needs further epidemiological studies (Mai and Morris 2013).

### Assessment of microbiome resilience

So far, there has been no consensus on the criteria for evaluating microbiome resilience, nor a standard approach. Limited studies measured microbiome resilience using genetic metrics, such as richness and evenness of gut bacteria, microbiome persistence degree, microbiome recovery rate. Shade et al. calculated the recovery rate of microbiome returning to its original parameters and exploited a resilience assessment formula (Shade et al. 2012). Hildebrand et al. calculated the microbiome persistence degree toward perturbations by comparing the metagenomic data from different timepoints in the same host (Hildebrand et al. 2021). We herein discuss assessment methods and potential parameters for evaluating microbiome resilience in both persistence and recovery.

### Assessment of microbiome structure and activities

#### Cultivation techniques

Cultivation is a conventional microbiological method used to isolate, propagate, identify, characterize, and manipulate microorganisms. Cultivation techniques address the limitations of metagenomic methods, enabling the investigation of bacterial phenotypic properties, beneficial or pathogenic activities, and therapeutic strategies. However, the weakness is the inability to study not-yet cultured microorganisms (Lee et al. 2021). A recent review article proposed the combination of traditional culture techniques with advanced genetic techniques to conduct in-depth analysis of gut microbiome at the strain level. The authors elaborated a variety of integrative techniques such as culturomics, droplet microfluidics, phenotypic and genomics selection, and membrane diffusion-based cultivation (Xu et al. 2024). Interestingly, all keystone species appear to be culturable, allowing for the establishment of strain-level libraries of gut keystone species. These libraries of comparative keystone culturomes allow the research of microbiome properties, dynamics, and resilient probiotic therapy.

#### Multi-omics analysis

Multi-omics is an integrated study to elucidate the structure, characteristics, dynamics, and functions of the microbiome by analyzing the metagenome, transcriptome, proteome, and metabolome (Lloyd-Price et al. 2019, Chetty and Blekhman 2024). Advanced technologies are often used in combination and, most recently, in conjunction with artificial intelligence (AI). Technology combinations have accelerated the research in microbiome and its resilience (Hernandez Medina et al. 2022, Chetty and Blekhman 2024). The strength of multi-omics analysis is that it provides high-throughput, accurate, and comprehensive data on microbiome profiling, community dynamics, and genetic properties, whereas the weakness lies in the complexity of data analysis and interpretation, lack of phenotypic information, and incomplete reference databases. The following are assessment methods and potential metrics for researchers and health services to evaluate microbiome resilience (Lozupone et al. 2012, Fassarella et al. 2021).

Microbiome diversity: A greater diverse gut microbiome tends to be more resilient to disturbances, while a decrease in microbiome complexity indicates a reduction in resilience (Lozupone et al. 2012, Palleja et al. 2018). Sequence-based microbiome data, for example, 16S rRNA gene sequencing data and multi-locus sequence typing (MLST) data, can be analyzed to compare the microbial diversity in normal or in response to different disturbances (Lozupone et al. 2012, Palleja et al. 2018, Bangayan et al. 2020). Microbiome diversity is evaluated on three scales: alpha diversity i.e. intra individual gut microbiome diversity, beta diversity i.e. inter-individual gut microbiome diversity within the same population, and gamma diversity i.e. species diversity between different populations (Mosca et al. 2016). It has been also suggested to use metagenomic sequencing to develop strain-specific metabolism pathways for comparative analysis (Tudela et al. 2021). Besides genome sequencing, flow cytometry techniques can be used to analyze gut microbiome composition based on flow cytometric signal variability (Esser et al. 2019). Recently, AI-powered techniques have been employed in processing and interpreting the vast polygenic data of gut microbiome (Hernandez Medina et al. 2022, Chetty and Blekhman 2024). This makes it possible to establish a correlation between microbiome resilience and microbiome diversity and allows researchers to monitor microbiome resilience by analyzing fluctuations in microbiome diversity.

Abundance of keystone species: Keystone species (explicated below) are essential for maintaining the homeostasis of the gut ecosystem. Scientists observed that the variation of these key members correlated to the dynamics and composition of the microbial community (Faith et al. 2013, Tudela et al. 2021). Increases or decreases in the abundance of keystone species or even absence, reflect the changes in microbiome resilience to stressors and health conditions (Palleja et al. 2018, Tudela et al. 2021, FitzGerald et al. 2022). Next-Generation Sequencing is a powerful tool to disclose the occurrence and relative abundance of the keystone species by detecting the key marker genes in stool samples (Tudela et al. 2021). In this way, *Akkermansia muciniphila* and *Faecalibacterium prausnitzii* were suggested as indicators for IBD, for their relative abundance significantly decreasing in ulcerative colitis and Crohn’s disease (Guo et al. 2021). Absolute abundance of the keystone species can be obtained using several methods, for example, culture-dependent assay, quantitative PCR by counting the copy numbers of keystone-specific genes, droplet digital PCR by detecting DNA variants in sample droplets with EvaGreen intercalating DNA dye, flow cytometry by counting and sorting microbes in fecal samples with fluorescent markers, and fluorescent in situ hybridization by visualizing and quantifying the bacteria with fluorescently labeled probes targeting keystone specific RNA sequences (Esser et al. 2019, Lee et al. 2021, Li et al. 2024).

Biomarkers here are regarded as measurable metabolites or proteins whose presence or absence indicates the status of microbiome (Llibre and Duffy 2018). Metabolic biomarkers can be used to observe the variation of microbiome, such as resilience and metabolic activities. Short-chain fatty acids (SCFAs) are outputs of gut bacteria and might serve as a biomarker for microbiome resilience (Salazar et al. 2022). Chronic inflammation has a negative impact on microbiome resilience. The levels of inflammatory biomarkers in the blood or stool have been used as the indicators of IBD, such as C-reactive protein, neutrophil–lymphocyte ratio, fecal calprotectin, stool lactoferrin (Guo et al. 2021). Pro-inflammatory cytokines, IgA antibodies, and antimicrobial peptides (AMPs), indirectly reflecting microbiome dynamic status, can be considered as potential biomarkers of microbiome resilience (Llibre and Duffy 2018). The integration of proteomics and genomics techniques sheds light on the development of biomarkers for microbiome screening and resilience assessment.

Recovery rate: the speed of the microbiome recovering from disturbances, representing the ability of the microbiome to respond to stressors. The recovery of gut microbiome after antibiotic exposure usually takes from a few weeks to several months (Palleja et al. 2018, Thriene and Michels 2023). Some researchers observed a prolonged recovery time for the gut microbiome fully returning to its original composition and diversity due to the factors as old age, low-fiber diet, long-term antibiotic use (Ng et al. 2019, Thriene and Michels 2023). It is worth noting that the gut microbiome can be constantly altered by disturbances, and some changes are irreversible (Dethlefsen and Relman 2011, Thriene and Michels 2023). To evaluate microbial community resilience, one approach is to measure the recovery rate of the microbial composition or functions after disturbances (Shade et al. 2012). Shade et al. developed a formula incorporating time to assess resilience, which calculated the rate of a microbiome returning to its original parameters. They determined the community was “recovered” when the means of parameters were statistically indistinguishable from the pre-disturbance means (Shade et al. 2012).

Persistence degree: the level of microbiome retention in response to environmental stressors (Hildebrand et al. 2021). Hildebrand et al. determined resilience as the degree of persistence and evaluated it by comparing strain-resolved metagenomic species (sMGS) at intervals. They calculated strain resilience using the number of consecutive timepoints with the same sMGS divided by the number of consecutive timepoints with different sMGS of the same host. The same algorithm was used to calculate the family persistence of strains shared between individuals in the same family (Hildebrand et al. 2021).

### Assessment of microbiome dynamics

Mathematical functions have been used to explore human microbiome dynamics *in vitro*. A previous study applied extended Taylor’s power law to determine spatial aggregation (i.e. heterogeneity) and temporal aggregation (i.e. stability) of the human microbiome at both population and community levels (Ma 2015). Nowadays, there has been a growing interest in using dynamic modeling approaches to explore the dynamics of microbial communities over time, particularly to track the fluctuations by external perturbations e.g. antibiotic exposure, lifestyle change, health status. Microbial dynamic modeling is an important tool to study microbiome resilience, which employs advanced computational algorithms to analyze metagenomic sequences, biochemical molecules, and genetic determinants in response to perturbations (Hernandez Medina et al. 2022). A recent review provided comprehensive insights into dynamic modeling systems exploited for studying small or large microbial communities using different molecular mechanisms at different scales (Qian et al. 2021). At ecological scales, Generalized Lotka-Volterra models describe how the absolute abundance of each community member changes with other community members as well as external inputs, while Data-driven dynamic regression models describe how community composition changes in response to an external input after being trained on a large volume of longitudinal data. At molecular effector scales, microbe-effector models describe how metabolites or immune molecules in the environment influence the growth of community members, while Genome-scale models describe the metabolic fluxes interacting with the growth rate of community members by using flux balance analysis and microbial genome scale metabolic networks (Qian et al. 2021). Today, machine learning and deep learning techniques are beginning to be used for examining microbe–microbe and host–microbe interactions at multiple omics levels, including the genome, transcriptome, epigenome, metabolome, and proteome. AI application can help predict the temporal dependencies and dynamic patterns of gut microbiome, allowing us to deeply understand microbiome behavior, resilience, and interactions (Hernandez Medina et al. 2022, Chetty and Blekhman 2024).

## Factors influencing microbiome resilience

The construction and succession of the microbiome begin at birth and continue throughout one’s life. A resilient microbiome, whether in the gut or other parts of the body, is typically composed of a highly diverse array of microorganisms (Lozupone et al. 2012, Rinninella et al. 2019, Dogra et al. 2020). Nevertheless, long-term disturbances, such as antibiotic use, can disrupt the microbiome structure leading to a decrease in microbial abundance and species richness, or even species loss (Jernberg et al. 2007, Jakobsson et al. 2010, Dethlefsen and Relman 2011, Palleja et al. 2018).

### Microbiome structure, activities, and interactions: determinants of resilience

#### Microbiome abundance and diversity

Sender et al. estimated approximate 38 trillion bacteria and 30 trillion human cells in an adult human body, while Rinninella et al. estimated over 100 trillion microorganisms in the human gastrointestinal tract (Sender et al. 2016, Rinninella et al. 2019). The colon is the most densely populated habitat for microbiome, containing 10^11^–10^12^ bacterial cells per milliliter (Rinninella et al. 2019). The gut microbiome encompasses large numbers of versatile microorganisms and complex environmental conditions across human populations, including trillions of bacteria, archaea, viruses, protists, and fungi. Amongst, the human gastrointestinal tract harbors a wide spectrum of bacteria, ranging from 200 to 1000 distinct species (Lozupone et al. 2012, Mosca et al. 2016, Rinninella et al. 2019). The gut microbiome forms a dense protective layer on the intestinal epithelium, resisting external pathogens invasion by competing for ecological niches, nutrients, and immune adaption (Lozupone et al. 2012). The microbiome structure varies among body parts, individuals, and populations, due to the specificities of habitats and niches, age, diet, geography, lifestyles etc. Around 1150 bacterial species were sampled from the gut of the European population, with an average of 160 species per person. In contrast, 1235 species-level phylotypes were recovered from the Chinese gut microbiome, assigned to 20 phyla, 36 classes, 72 orders, 121 families, and 290 genera, with an average of 186 ± 51 species-level phylotypes per person (Yang et al. 2020). Of the intestinal bacteria, 99% are anaerobic bacteria, and 90% belong to the phyla Bacillota and Bacteroidota. Bacillota mainly consists of Gram-positive bacteria, like *Bacillus, Clostridium*, and *Streptococcus*, while Bacteroidota mainly consists of Gram-negative bacteria, like *Bacteroides* and *Prevotella* (Xue et al. 2020). A diverse microbiome has a strong capacity to resist perturbations and rapidly recover from microbial imbalances (i.e. resilience), thus favoring the host health (Lozupone et al. 2012, Rinninella et al. 2019, Philippot et al. 2021). Researchers observed the low-diversity microbiome accompanied by loss of symbionts or emergence of pathobionts was associated with dysbiosis and chronic inflammatory diseases (Guo et al. 2021, Malard et al. 2021, Weiss et al. 2021). For example, the longitudinal studies of IBD revealed the decreased resilient microbiome coupled with the microbiome shifting toward aerotolerant pro-inflammatory clades in IBD (Lloyd-Price et al. 2019, Guo et al. 2021). The mechanisms employed by diverse microbes to steer the microbiome resilience are discussed here: (1) Heterogeneous microbial taxa perform a wide range of functions, including metabolizing nutrients, resisting pathogens, and modulating the immune system; (2) a diverse microbial consortium provides functional redundancy, which helps buffer against functional aberrations in the ecosystem caused by the loss of taxa due to environmental stressors; (3) different types of bacteria regulate host immunity via different pathways (Wagg et al. 2019, Biggs et al. 2020). Some bacterial groups have been highlighted as keystone species, which play a pivotal role in retaining microbiome structure and functions.

#### Keystone species

Keystone species, originally proposed in ecology, are defined as “*the taxa which have major influence on microbiome composition and function at a particular space or time*” (Banerjee et al. 2018). They can be animals, plants, or microorganisms. Keystone species determinate microbiome resilience and manipulate the whole ecosystem, for their big and disproportionate impact on the overall structure and functionality of the microbiome (Mouquet et al. 2013). *Bacteroides, Akkermansia*, and *Faecalibacterium*, for instance, were regarded as keystone genera because they are closely associated with gut microbiome variations and host health (Guo et al. 2021, Tudela et al. 2021). Keystone species are not necessary to be the dominant components of a microbiome; they can be identified in high or low abundance. Herren and his colleagues concluded from all datasets that the bacterial taxa ranked in abundance and the taxa ranked in connectedness were positively, but not strongly correlated. Some low-abundant keystone species were the best predictors of changes in the microbiome composition of the entire community (Herren and McMahon 2018, Guo et al. 2021). This indicates that certain keystone species often have an over-proportional impact on ecosystem compared to their numbers (Faith et al. 2013, Banerjee et al. 2018). Based on the literature, we provide an overview of a few prominent keystone species and their functional activities in the gut ecosystem (Shetty et al. 2017, Tudela et al. 2021).

- *Akkermansia muciniphila*: a species of Gram-negative anaerobic bacteria belonging to the genus *Akkermansia*. They are regarded as beneficial commensal bacteria that colonize the human intestinal mucosa and act as a barrier protecting the gut lining. They also have a substantial function of mucin degradation (Bae et al. 2022).
- *Bacteroides thetaiotaomicron*: a species of Gram-negative, anaerobic bacteria belonging to the genus *Bacteroides*. They are abundant in the human intestinal tract, and they have the capabilities of complex carbohydrates, e.g. arabinogalactan degradation and selective bile salt hydrolase (BSH) activity (Porter et al. 2018).
- *Bifidobacterium species:* several species of Gram-positive anaerobic bacteria belonging to the genus *Bifidobacterium*. Bifidobacteria are highly present in the healthy infant gastrointestinal tract, with *B. longum, B. breve*, and *B. bifidum* generally being dominant species in infant. However, the levels of bifidobacteria decrease considerably with age, remain relatively stable at a low level throughout adulthood, and decrease again at old age. The species composition also shifts, with *B. catenulatum, B. adolescentis*, and *and B. longum* being dominant bifidobacteria in adults (Arboleya et al. 2016). Bifidobacterial species have health-promoting activity. *Bifidobacterium pseudolongum* can break down complex carbohydrates. *Bifidobacterium longum* is capable of degrading carbohydrates particularly Human Milk Oligosaccharides and has BSH activity (Picard et al. 2005). Therefore, *Bifidobacterium* sp. is frequently employed as probiotic ingredients in many functional foods, and these probiotic supplements play a beneficial role in improving the health conditions of adults and elderly.
- *Christensenella minuta:* a newly identified species of Gram-negative, anaerobic, and nonmotile bacteria. They have the activities of stimulating ecosystem diversity and producing acetate and BSH activity (Kropp et al. 2021).
- *Faecalibacterium prausnitzii:* a species of Gram-positive, mesophilic, anaerobic bacteria that are one of the most abundant commensals of the human gut microbiome. They can produce butyrate and metabolites with anti-inflammatory effects (Lopez-Siles et al. 2017).
- *Methanobrevibacter smithii:* a dominant archaeon comprising up to 10% of anaerobes in the colons of healthy adults. They can produce acetate and methane from hydrogen (Samuel et al. 2007).
- *Ruminococcus bromii:* a species of Gram-positive anaerobes and a dominant member of the human gut microbiome. They have the activities of releasing energy from degrading starches especially “resistant” starch and produce butyrate (Ze et al. 2012).

Microbial activities of keystone species help shape gut microbiome, including flexible metabolism, antimicrobial-agents production, quorum sensing, horizontal gene transfer, and host immune modulation. Mucin degradation capability provides energy and nutrient sources of carbon and nitrogen for bacterial survival and growth (Glover et al. 2022). Many keystone taxa possess mucin-degrading glycosyl hydrolases, indicating these microbes have mucin-degradation potential. Metabolism of mucin O-linked glycans facilitates the adhesion of bacterial species to the gut mucus layer, which is a key step in microbiome establishment particularly in early life (Chng et al. 2020, Glover et al. 2022). However, excessive mucin degradation by mucolytic bacteria is considered an initial stage of pathogenesis, since it undermines the protection of the host mucus layer (Glover et al. 2022). The keystone species also produce metabolites, such as SCFAs, bacteriocin, hydrogen peroxide, AMPs, and lysozymes that can boost colonization/multiplication of commensal bacteria and suppress/eliminate pathogenic bacteria (Liu et al. 2022). These metabolic products can be classified into three categories based on their activities: (1) Nutritious substances e.g. carbohydrates, amino acids, and vitamins, which can be employed for interspecies cross-feeding and gut microbiome rebuilding; (2) signaling molecules, which conduct cross-talking between microbes and between microbes and their host; (3) bactericidal substances, which inhibit or eliminate of exogenous pathogens. SCFAs are acetate, propionate, and butyrate derived from bacteria fermenting dietary fibers (Fusco et al. 2023). They are released in large amounts in the proximal colon at approximately 130 mmol per kilogram of luminal contents, and serve as energy suppliers, pH regulators, epithelium strengthener, and immunomodulators for host defense (Shimotoyodome et al. 2000, Parada Venegas et al. 2019, Lo et al. 2021, Fusco et al. 2023). *Lactobacillus* sp. and *Bifidobacterium* sp. were found to activate Paneth cells to secrete regenerating islet-derived protein III-gamma (RegIIIγ), which has bactericidal activity toward pathogenic bacteria (Shin et al. 2023). Keystone species also act as major colonizers to facilitate microbiome post-antibiotic recovery (Tudela et al. 2021). Long-term disturbances were found in the reduction or even loss of several keystone species, ultimately leading to microbiome resilience declining (Palleja et al. 2018, FitzGerald et al. 2022).

#### Microbe–microbe interplays

Microbial interplay is significant for achieving homeostasis and functionality of the microbiome through coordinated responses between microorganisms. Literature overviews the types of microbe–microbe interplays within various habitats and their impacts on the microbiome dynamics and resilience (Nagaoka et al. 2008, Mosca et al. 2016, Fassarella et al. 2021).

- Mutualism refers to co-dependent participants benefiting from each other and increasing physical fitness. Co-digestion of complex carbohydrates and the synthesis of essential vitamins provide energy and nutrition for microbial adaption and succession. Bacterial coaggregation is the process by which different species or strains adhere to one another. Gut bacterial coaggregation facilitates forming complex multi-species communities by recognizing homologous surface components of genetically distinct microorganisms and binding each other.
- Cooperation refers to non-obligatory co-dependence symbionts working together. It renders microorganisms collectively responses to environmental perturbations in order to adapt to changes by, for example, quorum sensing and cross-feeding.
- Commensalism refers to one partner getting the advantage without any help or harm to the other. It provides microbial colonization and growth with provision of nutrients and protection, as well as modulation of host immune tolerance. Bacterial co-adhesion refers to the process by which bacteria adhere to surfaces with the help of other bacteria. For example, in oral cavities, the adhesion of *Bifidobacterium adolescentis* to tooth surfaces is mediated by the coaggregation of oral bacteria (Nagaoka et al. 2008, Mosca et al. 2016, Fassarella et al. 2021).
- Amensalism refers to one microorganism being inhibited or destroyed, and the others are unaffected. It prevents the colonization and invasion by pathogens through the antimicrobial agents produced by some bacterial species.
- Competition refers to both the participants harming each other for resources and space. It selects the best-adapted species/strains that colonize the mucosal surface of epithelial lining through specific adhesion mechanisms.
- Parasitism refers to one organism getting benefited at the expense of the other. It gives selective pressure to microbiome members and changes to microbial composition.
- Predation refers to one microorganism devouring or attacking the other, leading to harm or death to the latter. It promotes microbiome complexity by exerting selective pressure and by releasing nutrients through the lysis of bacterial cells by lytic bacteriophages.

In brief, microbe–microbe interplays determine the microbiome resilience with respect to microorganism reproduction and colonization, microbial community shaping and rebuilding through the activities of cross-feeding, quorum sensing, co-adhesion and coaggregation, and cell–cell competing and attacking. Long lasting disturbance disrupts the coordination of gut microbes and results in microbiome destruction, resilience reduction, and a number of health problems (Mosca et al. 2016, Palleja et al. 2018, Frost et al. 2021, Malard et al. 2021).

### Host–microbe interactions: driving force of resilience

**Figure 2.**
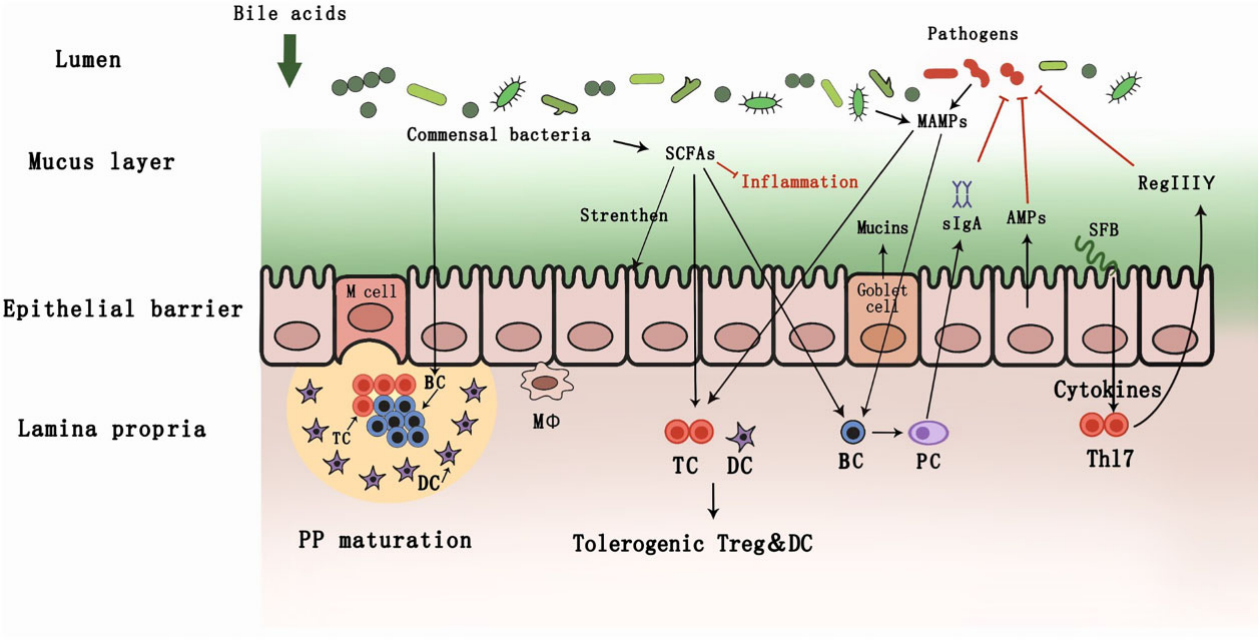
Host–microbe interactions in association with microbiome resilience: immune facilitating microbial preservation, colonization, construction, protection, and suppression via mucins, Treg, M_Ø_, RegIIIγ, sIgA, AMPs, and microbial immunomodulation via LPSs, SCFs, and MAMPs.

The digestive tract is the largest immune organ in the human body and is the most densely populated habitat for microbes. It consists of the cells from non-hemopoietic origin (e.g. epithelia, Paneth cells, goblet cells), hemopoietic origin (e.g. macrophages, dendritic cells, lymphocytes), and microbial origin (e.g. bacteria, fungi, protozoa, virus) (Chassaing et al. 2014). Gut-associated lymphoid tissues (GALTs) contain up to 70% of the body’s immune cells spreading in Peyer’s patches (PPs), the vermiform appendix, and the isolated lymphoid follicles across the small and large intestines (Morbe et al. 2021, Wiertsema et al. 2021). Microbes and the host immune system have been coevolving for millions of years to achieve a homeostatic symbiosis. At birth, the fetal gut is exposed to numerous microorganisms. The early resident bacteria engage in inducing, training, and regulating the host immune system (Belkaid and Hand 2014, Chassaing et al. 2014). On the other hand, TLR5 expressed by dendritic cells, primarily recognizes bacterial flagellin manipulates gut microbiome formation in the neonatal period. The composition of neonate microbiome determines immunomicrobial homeostasis and microbiome resilience in later life (Zheng et al. 2020). The resilience of the gut microbiome fluctuates along with host age, being lowest in infancy, peaking in adulthood, and declining in old age (Badal et al. 2020, Schwartz et al. 2020, Bosco and Noti 2021). Declined host immunity and reduced resilient microbiome contribute to a variety of health conditions and diseases in the elderly (Thevaranjan et al. 2018, Dillon et al. 2020, Walrath et al. 2021). Studies discovered that IBD, coeliac disease, and systemic diseases (autoimmune diseases like rheumatic arthritis and systemic lupus erythematosus, metabolic diseases, and heart diseases) often accoupled with intestinal immunity dysfunction and microbiome dysbiosis (Chassaing et al. 2014, Liu et al. 2018, Yoo et al. 2020, Zheng et al. 2020). Unlike other articles focusing on immunomodulation by the microbiome, we highlight the role of the host’s immune system in regulating the gut microbiome, especially microbiome resilience. The strategies employed by the host immune system to regulate microbiome resilience include (1) selecting resident commensals and supporting their reproduction; (2) rewarding the cooperative interactions of symbiont strains and punishing the non-symbiotic interactions; and (3) reducing the direct conflicts between different symbionts in a single host (Chomicki et al. 2020). Impaired or aberrant immune responses can lead to local or systemic bacterial dissemination and increasing risk of infections. For example, the unique fecal microbiome with low species richness was identified in HIV-infected patients, making them more susceptible to various infections (Fung et al. 2014, Ribeiro et al. 2017). The involvement of the host immune system in microbiome resilience is discussed regarding immunological tolerance, microbiome compartmentalization, immune inclusion and exclusion, and microbial immunomodulation (Fig. 2) (Dantzer et al. 2018, Lo et al. 2021).

#### Immunological tolerance

Immunological tolerance refers to the suppression of inflammatory responses to certain microorganisms and foods, which is likely achieved through multiple and redundant microbial-immune mechanisms (Belkaid and Hand 2014). It arises from the first encounter with a specific antigen and continues throughout life. Immunological tolerance maintains the gut microbiome through preserving commensal microorganisms from harmful immune responses. Harmful immunity can be induced by pathogenic organisms and their fractions or by benign antigens from commensals or food. When the immune tolerance in the intestine fails, a range of health problems appear, from IBD to allergies (Belkaid and Hand 2014). Immunological tolerance assists the establishment of the gut microbiome from 0 to 3 years as well as microbiome repair and recovery throughout the lifespan. The formation of Treg cells and the subsequent production of IL-10 are vital for mediating immune tolerance. *Bacteroides fragilis*, a key species of gut microbiome, was observed to colonize the colon under IL-10-driven immunotolerance (Round and Mazmanian 2010). Upon recognizing capsular polysaccharide A on *B. fragilis*, lamina propria dendritic cells were activated through Toll-like receptor (TLR) 2-dependent pathway and then presented the antigen to naive CD4^+^ T cells. In the presence of activated transforming growth factor (TGF)-β, the naive T cells differentiated into Treg cells and produced IL-10 (Mazmanian et al. 2005, Zheng et al. 2020). For another example, the presence of indigenous clostridial consortium in the cecum and proximal colon (e.g. *Clostridium* clusters IV and XIVa) was found to be associated with Treg mediated tolerance (Belkaid and Hand 2014). The development and functions of immunological tolerance are multiple, complex, and redundant. The foremost mechanism of tolerance generation involves a cluster of differentiation 4 (CD4^+^) forkhead box protein 3 (FOXP3^+^) regulatory T cells (Tregs) and their inhibitory activities (Round and Mazmanian 2010, Belkaid and Hand 2014, Mowat 2018). Tregs are a specialized subset of T cells that can be induced both in thymus and in gut and then migrate to the lamina propria via efferent lymph and the bloodstream. CD4+ FOXP3+ Tregs can suppress the immune responses that are elicited by other immune cells. The functions of CD4+ FOXP3+ Treg-mediated tolerance include (1) to inhibit the proliferation of various T cell types through attenuating IL-2 production, (2) to suppress the activation of effector T cells through expressing cytotoxic T lymphocyte antigen IV (CTLA-4), (3) to inhibit antigen-presenting cells and T cells through producing cytokines IL-10 and TGF-β, and (4) to induce cytolysis through secreting Granzyme A or B and perforin (Round and Mazmanian 2010, Mowat 2018, Grover et al. 2021, Akagbosu et al. 2022). IL-10 is an anti-inflammatory cytokine produced by local mucosal macrophages or circulating plasma cells and that facilitates Treg cell survival and activity (Mowat 2018). Another mechanism of immunological tolerance generation is mediated by tolerogenic dendritic cells, a subset of dendritic cells with immuno-suppressive activities (Mowat 2018, Akagbosu et al. 2022). The immunosuppressive activities of tolerogenic dendritic cells include (1) to inhibit effector T cell proliferation and response, (2) to induce Treg cell generation, (3) to activate anergic T cells, and (4) to foster redundant tolerogenic dendritic cells (Mowat 2018). These tolerance activities can be regulated by immunomodulatory molecules (e.g. IL-10, TGF-β, programmed death-ligand-1/2, CTLA-4, and immunoglobulin-like transcript-3/4) produced in the expression of other death receptors and the deprivation of nutrition factors etc. (Domogalla et al. 2017). The third mechanism is that early exposure to bacteria or bacterial derivatives such as sphingolipids, leads to the repression of invariant natural killer T cells (iNKTs), thereby allowing the retention and inhabitation of certain microbes (Belkaid and Hand 2014). iNKTs are a specialized T cell population, phenotypically and functionally resembling NK cells or memory T cells. They have the abilities of cytokine production and cytotoxicity (Hapil and Wingender 2018). Immune tolerance mediated by tolerogenic Tregs, dendritic cells, or suppressive iNKTs, facilitates bacterial survival, colonization, and propagation, especially under the adverse conditions such as disorders.

#### Immune-driven microbiome compartmentalization

Microbiome compartmentalization refers to the segmental distribution and spatial organization of microorganisms in distinct ecological niches or habitats. Microbiome compartmentalization involves microbial selection, attachment, and community establishment (Belkaid and Naik 2013). Bacteria have their own tropism, for example, Actinomycetota, Bacillota, and Pseudomonadota predominating in the skin, Bacillota predominating in the vaginal tract, and Bacteroidota, Bacillota, Fusobacteria, and Pseudomonadota predominating in the digestive tract. Microbiome compartmentalization is driven by multi-factors, such as tissue-specific cells, mucus, O_2_ and pH gradients, nutrient supplements, antimicrobial agents, and immune modulation (Belkaid and Naik 2013, Fung et al. 2014, Canesso et al. 2022). Gut epithelial cells and their secretions direct the attachment between microorganisms and host tissues (Okumura and Takeda 2017). In the stomach and upper intestine, bile acids lower the occurrence and number of resident microorganisms (Hooper et al. 2012). Mucins secreted by goblet cells act as a barrier limiting the direct contact between bacteria and enteric cells by forming a layer between the intestinal epithelium and the lumen (Johansson et al. 2011, Okumura and Takeda 2017). Commensal bacteria attach to the outer mucus layer, and they cannot cross the inner mucus layer. Mucoprotein 2, secreted primarily by colonic goblet cells, is the most abundant mucin component in the colon. Its structure O-glycans facilitate the attachment of microbes in the colon by serving as binding sites for microbial adhesion (Johansson et al. 2011). The attached bacteria subsequentially co-aggregate with the other bacteria to establish a microbial community. AMPs are secreted by primarily Paneth cells located in the small intestine and other immune cells such as neutrophils and macrophages. Some AMPs expressions require the engagement of pattern recognition receptors (PRRs) driven by commensal derivatives, while others, such as α-defensins, can be constitutively secreted by epithelial cells (Belkaid and Hand 2014). AMP compounds exert functional activities of bactericidal, anti-inflammation, and anti-endotoxicity that affect microbial community construction. The mechanisms of antibacterial activities include metal ion chelation, enzymatic degradation of bacterial cell membrane, and modulation of innate immune response after binding to bacterial lipopolysaccharides (LPSs) (Fung et al. 2014). Immune cells also direct microbiome compartmentalization, for example, macrophages together with T and B cells restricting the commensal translocation and dissemination (Belkaid and Hand 2014). When commensal bacteria translocate across the enteric epithelial cells, they can be rapidly engulfed and eliminated by macrophages who reside in the lamina propria, or they can be captured alive by dendritic cells and then traffic to the mesenteric lymph nodes via the intestinal lymphatics (Belkaid and Hand 2014, Fung et al. 2014). Bacteria-loaded dendritic cells can induce the differentiation of commensal-specific Th17 cells and Immunoglobulin A (IgA)-secreting B cells. Transcytosis IgA limits microbial translocation by regulating bacterial gene expression and inhibiting their attachment to the epithelial surfaces (Abokor et al. 2021). Although IgA antibodies are essential for gut microbiome, the functions of IgAs produced in different GALT compartments and their action pathways in response to different intestinal microorganisms remain unclear. In brief, immune-driven microbiome compartmentalization involves microbial attachment, distribution, and community construction, all of which correspond to microbiome resilience.

#### Immune inclusion and exclusion

As mentioned above, epithelial cells and their secretions constitute the first line of innate immune defense against external microbial colonization and intrusion. Host immunity is initiated in response to the presence of foreign bodies. Immune cells (e.g. monocytes and macrophages) recognize microorganisms by pattern-recognition receptors (PRR, e.g. TLRs) through binding to microorganism-associated molecular patterns (MAMPs, e.g. peptidoglycan, LPSs, bacterial DNA) (Willing et al. 2010, Wiertsema et al. 2021). The recognition of non-self-microorganisms triggers rapid innate responses, including phagocytosis and microbicidal activation, inflammatory responses, and complement activation (Chaplin 2010, Belkaid and Hand 2014). Adaptive immunity constitutes the second line of host’s defense, including antigen-specific T and B lymphocytes. They provide specific responses to external and internal microbes (Belkaid and Naik 2013, Canesso et al. 2022). The process of cellular immunity goes as follows: (1) activating macrophages and NK cells to destroy intracellular bacteria, (2) activating cytotoxic T cells to kill the pathogens infected body cells, and (3) secreting cytokines to orchestrate host immune responses (Chaplin 2010, Zheng et al. 2020). Humoral immunity specifically targets, neutralizes, optimizes, and eliminates the extracellular pathogens through antibody secretion by plasma cells and complement cascade activation (Chaplin 2010, Belkaid and Harrison 2017). In GALTs, Treg-produced TGF-β triggers isotype IgA switching from the class of immunoglobulins via either T cell-independent or T cell-dependent pathway (Zheng et al. 2020, Abokor et al. 2021). PPs are the major site where antibody-secreting plasma cells are induced and produce secretory IgA antibodies (sIgAs). In intestinal homeostasis, sIgAs get bound to commensal bacteria and keep them thriving (Abokor et al. 2021, Takeuchi and Ohno 2021). In intestinal disorders, sIgAs help intestinal dendritic cells select symbiotic strains and protect sIgA-coated commensals from being attacked (Belkaid and Hand 2014, Abokor et al. 2021). SIgA-microbiome interactions are classified as (1) cross-species interaction, sIgAs binding to a broad subset of microbiome, typically to structurally distinct antigens like lipopolysaccharide, cytosine-phosphor-guanine; (2) species-specific interaction, sIgAs binding exclusively to distinct bacterial species, for example, to bacterial surface carbohydrate moieties; (3) strain-specific interaction, sIgAs binding selectively to various genetic variants or subtypes within a bacterial species (Abokor et al. 2021). Besides the protective role, sIgAs, as a key member of humoral mucosal immunity, can also inhibit or exclude enteropathogenic bacteria (Belkaid and Hand 2014, Takeuchi and Ohno 2021). The bacterial exclusion mechanisms of sIgAs include (1) to limit pathogen attachment and invasion of host intestinal epithelia through sIgA coating and agglutination (2) to reduce bacterial unchained growth by inhibiting bacterial conjugation, and (3) to promote bacterial selection through antigen sampling by activated dendritic cells in PPs (Xue et al. 2020, Abokor et al. 2021, Takeuchi and Ohno 2021). Without PRR-mediated bacterial sensing, the class of immunoglobulins switches to other types of antibodies, such as anti-commensal IgG, but not sIgA (Chaplin 2010, Hooper et al. 2012). Elevated levels of systemic and local anti-commensal IgG and auto-antibodies were observed in human IBD (Castro-Dopico and Clatworthy 2019).

#### Microbial immunomodulation

Gut microbes are involved in regulating the development and function of the immune system, thus indirectly affecting microbiome resilience. Studies have found microbiome imbalance can impair gut immunity and increase the risk of intestinal disorders and systemic or metabolic diseases (Zheng et al. 2020).

The development of GALTs has been demonstrated to be associated with gut microbiome (Belkaid and Hand 2014, Xue et al. 2020). Isolated lymphoid follicles are induced and develop after exposure to microorganisms at birth (Belkaid and Hand 2014). The development and maturity of PPs and mesenteric lymph nodes are also achieved in response to microorganisms (Xue et al. 2020). Moreover, the generation and differentiation of T lymphocyte subsets in the lamina propria are subjected to bacterial species that engage in regulating local and systemic immune responses (Hooper et al. 2012, Belkaid and Hand 2014). Germ-free animals experience serious defects in their immune network of organs, immune cells, immune proteins, and chemicals, such as abnormality of αβ and γδ intra-epithelial lymphocytes, early B cell lineage in the intestinal mucosa, IgA antibodies, immunomodulatory effector cells, and effector T lymphocytes. These defects can be reversed after microbiome inoculation (Xue et al. 2020, Zheng et al. 2020).

Host immune response also relies on sensing and responding to microorganisms, microbial fractions, or derivatives in the gut. *Lactobacillus* sp. was found to induce the secretion of mucins in the gut, which formed a protective layer inhibiting the adhesion of pathogenic *Escherichia coli* (Qin et al. 2022a). *Clostridium* sp., a large group of Gram-positive anaerobes, were identified as the potent inducers of Treg cells (Abokor et al. 2021). *Clostridium* sp. and their derivative butyric acid induced the expression of TGF-β and FOXP3 facilitating the proliferation and differentiation of FOXP3^+^ Treg cells (Xue et al. 2020). The role of FOXP3^+^ Treg cells in mediating immunological tolerance has been addressed above. Commensal-specific Tregs can promote IgA^+^ B cell class-switching and suppress excessive mucosal inflammation (Hooper et al. 2012, Belkaid and Hand 2014). In murine studies, segmented filamentous bacteria (SFB) were found to activate the differentiation of functionally distinct Th17 cells and the production of IL-17 and IL-22 (Willing et al. 2010, Hooper et al. 2012, Zheng et al. 2020). SFB-activated Th17 cells produced non-inflammatory AMPs, whereas Citrobacter-activated Th17 cells produced inflammatory cytokines (Zheng et al. 2020). Bacteria-activated Th17 cells produced cytokines IL-17 and IL-22 that exhibited divergent pro-inflammatory and regenerative properties (Belkaid and Hand 2014, Zheng et al. 2020). Microbial-induced IgA synthesis is associated with two tumor necrosis factors (TNFs), B cell activating factor and proliferation-inducing ligand (APRIL). Upon sensing commensal bacteria by TLRs, the intestinal epithelial cells release APRIL that induces IgA class switching. Studies have found the defects in microbiome-IgA axis led to the occurrence of various diseases, such as colitis, colorectal cancer, and nephropathy (Zheng et al. 2020, Abokor et al. 2021). A recent publication summarizes the contribution of intestinal keystone species to the host immune system (Xue et al. 2020).

- *Bacteroides thetaiotaomicron* and *Faecalibacterium prausnitzii* promote goblet cell differentiation and stimulate mucus secretion.
- *Lactobacillus casei* triggers the humoral immunity response and limits excessive immune response by increasing sIgA concentration and elevating the expression of FOXP3.
- *Bifidobacterium* sp. plays a crucial role in maintaining gut immune homeostasis by alleviating intestinal inflammation, mediating humoral immunity, and dampening excessive immunity. The mechanisms include promoting the differentiation of T helper cells, stimulating the secretion of IL-1 and IL-6, reducing the lipopolysaccharide-induced damage, inhibiting proinflammatory cytokine secretion, increasing sIgA concentration, and the expression of FOXP3.
- *Bacteroides fragilis* enhances the type 1 immunity and accelerates the differentiation of Treg cells by producing polysaccharide A, which has been shown to induce the production of type 1 cytokines such as IFN-γ and IL-2.

A variety of bacterial fractions also induce innate immune response via different TLR signaling pathways, such as the myeloid differentiation primary-response protein 88 (MYD88) signaling pathway (Kawai and Akira 2010). LPSs, the components of the Gram-negative cell wall, primarily activate the TLR4 signaling pathway. This leads to intestinal epithelium proliferation and epithelial barrier integrity. Flagellins, the component proteins of bacterial filaments, activate the TLR5 signaling pathway, which elicits epithelial AMP production (Kawai and Akira 2010, Hooper et al. 2012, Zheng et al. 2020). The production of AMP through TLR5 signaling pathway is as follows: (1) Dendritic cells producing TLR5 in the lamina propria, (2) flagellin-TLR5 stimulating the production of IL-23, (3) IL-23 promoting the expression of IL-22 by innate lymphoid cells, and (4) IL-22 inducing Th17 cells to produce AMP RegIIIγ (Hooper et al. 2012). Th17 cells represent a class of potent immunomodulatory effectors that are present in high numbers in the lamina propria of small intestines. The IL-22-IL-23 pathway involves gut epithelial repair and regeneration, protective and inflammatory immunity balance, and microbiome shaping. Inactivation of IL-23-IL-22 signaling pathway has been linked to intestinal barrier damage, dysbiosis, and the spread of pathogenic bacteria (Fatkhullina et al. 2018).

In conclusion, microbiome resilience is an intrinsic capability to maintain homeostasis in an ecosystem. Exploring microbiome resilience helps us understand how the gut microbiome responds to various stressors and maintains or restores balance. Resilience assessment can be used as a tool to monitor human health status and to evaluate the outcomes of drug and immunomodulation therapies.
